# Diversity when interpreting evidence in network meta-analyses (NMAs) on similar topics: an example case of NMAs on diabetic macular oedema

**DOI:** 10.1186/s13643-023-02349-4

**Published:** 2023-10-07

**Authors:** Jing Wu, Clive Adams, Xiaoning He, Fang Qi, Jun Xia

**Affiliations:** 1 School of Pharmaceutical Science and Technology, Tianjin University, Nankai District, No. 92 Weijin Road, Nankai District, Tianjin, CO 300072 China; 2 Center for Social Science Survey and Data, Tianjin University, Tianjin, China; 3 Mental Health Services Research, University of Nottingham, Nottingham, UK; 4 Academic Department, Systematic Review Solutions Ltd, Shanghai, China; 5 The Nottingham Ningbo GRADE Centre, The University of Nottingham Ningbo, Ningbo, China; 6 Division of Epidemiology and Public Health, School of Medicine, University of Nottingham, Nottingham, UK

**Keywords:** Diabetic macular oedema, Aflibercept, Ranibizumab, Network meta-analysis, Critical appraisal

## Abstract

**Background:**

Different network meta-analyses (NMAs) on the same topic result in differences in findings. In this review, we investigated NMAs comparing aflibercept with ranibizumab for diabetic macular oedema (DME) in the hope of illuminating why the differences in findings occurred.

**Methods:**

Studies were searched for in English and Chinese electronic databases (PubMed, Embase, Cochrane Library, Web of Science, CNKI, Wanfang, VIP; see detailed search strategy in the main body). Two independent reviewers systematically screened to identify target NMAs that included a comparison of aflibercept and ranibizumab in patients with DME. The key outcome of interest in this review is the change in best-corrected visual acuity (BCVA), including various ways of reporting (such as the proportion of participants who gain ≥ 10 ETDRS letters at 12 months; average change in BCVA at 12 months).

**Results:**

For the binary outcome of BCVA, different NMAs all agreed that there is no clear difference between the two treatments, while continuous outcomes all favour aflibercept over ranibizumab. We discussed four points of particular concern that are illustrated by five similar NMAs, including network differences, PICO (participants, interventions, comparators, outcomes) differences, different data from the same measures of effect, and differences in what is truly significant.

**Conclusions:**

A closer inspection of each of these trials shows how the methods, including the searches and analyses, all differ, but the findings, although presented differently and sometimes interpreted differently, were similar.

**Supplementary Information:**

The online version contains supplementary material available at 10.1186/s13643-023-02349-4.

## Background

With the rapid growth of biomedical evidence, systematic reviews provide an opportunity to make healthcare decisions based on comprehensive syntheses of the best available evidence on a topic [1–3]. Current knowledge may be imperfect, but decisions should be better informed when made in the light of the best, most up-to-date knowledge. It is essential that the systematic review itself is both clear and accurate for local interpretation by healthcare decision-makers (healthcare practitioners and policy-makers) [3, 4]. A problem arises when different research teams use similar approaches to synthesise evidence but report conflicting results. In this review, we investigate one such example in the hope of shedding light on the reasons for the differences in findings.

Our example comes from network meta-analyses (NMAs) comparing aflibercept (a vascular endothelial growth factor inhibitor) with ranibizumab (a monoclonal antibody) for diabetic macular oedema (DME). Diabetic retinopathy (DR) is a common complication of diabetes and the leading cause of blindness in the population of working age [5], with DME present in 4–7% of the population with DR [6]. DME impairs vision-related functioning and quality of life (QoL) [7]. It is the leading cause of moderate to severe visual impairment in the population with diabetes. DME is a significant economic burden for patients and public health systems [8, 9]. The therapeutic goal for people with DME is to improve visual function and vision-related QoL [10]. Anti-vascular endothelial growth factor (VEGF) is recommended as a first-line treatment in several clinical guidelines [11, 12]. Aflibercept and ranibizumab are commonly used in clinical practice, but direct comparisons of these two drugs are limited. NMA then becomes an attractive option, as NMAs use previous studies of the two drugs directly compared with other controls to create statistically indirect comparisons of the two drugs of current interest.

## Methods

We searched English and Chinese electronic databases (PubMed, Embase, Cochrane Library, Web of Science, CNKI, Wanfang, VIP (with search strategies described in Additional file 1)). Two authors (XNH and FQ) independently screened the search results to identify target NMAs that included a comparison of aflibercept and ranibizumab in patients with DME. For each included NMA, two authors (XNH and FQ) independently extracted the general study characteristics (such as search databases, number of studies, PICO [participants, interventions, comparators, outcomes] information) and assessed the quality of the included NMAs using the AMSTAR-2 tool [3]. Disagreements at each stage were resolved by author team discussion and consensus. Tables and figures were used to summarise and present descriptive information.

## Results

**Fig. 1:**
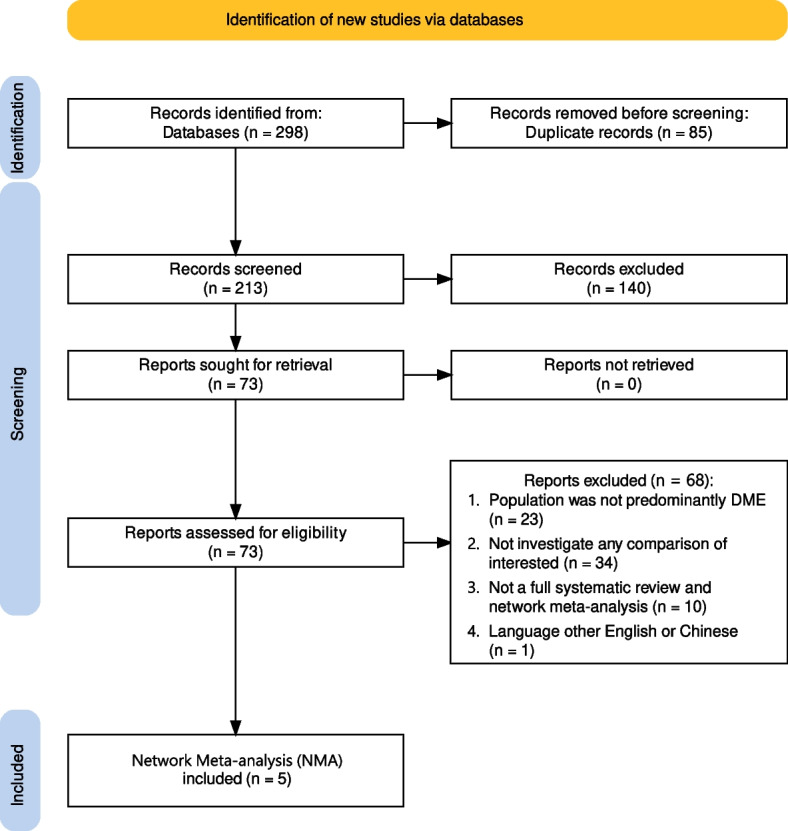
Flow diagram for identification of new studies via databases

We finally identified five NMAs [13–17], and the screening processes are summarised in Fig. 1.

### General study characteristics

Everything seemed varied in almost every NMA (Table 1). Although the question under investigation was consistent, the searches, the numbers of trials used, and the definitions for eligible participants, comparisons from which to source data and acceptable outcomes mostly lacked rigid consistency. The work for these NMAs spanned at least 5 years based on the search dates (2013 to 2017). All included NMAs searched three main databases (MEDLINE/PubMed, EMBASE, and Cochrane Library). Korobelnik et al. [13] and Régnier et al. [14] additionally searched abstracts and unpublished data, and Muston et al. [16] and Virgili et al. [17] additionally searched other clinical trial registration platforms. The main difference in the eligibility criteria is the inclusion of interventions (such as bevacizumab, triamcinolone acetonide or pegaptanib was not in all NMAs). Therefore, the inconsistent inclusion of trials in the NMAs was due to number of reasons, the most important being the scope of the search and the inclusion criteria (such as the dosage of interventions, the regimen [PRN (pro re nata) or T&E (treat and extend)]). Details of the included trials in these NMAs and the situation of overlap are shown in Additional file 2. Despite lacking a rigid consistency of definitions, study identification and sampling across the five NMAs, the participants, studies, and outcomes were, generally speaking, recognisable to those with clinical and academic experience in this field.

**Table 1:** Summary of Included NMAs

			**Study ID**^**a**^				
			Korobelnik 2015 [13]	Régnier 2014 [14]	Zhang 2016 [15]	Muston 2018 [16]	Virgili 2018 [17]
**Protocol identified**			x	x	x	x	√
**Search**	Cochrane		√	√	√	√	√
	EMBASE		√	√	√	√	√
	MEDLINE		√^b^	√^b^	√^c^	√^b^	√
	Others		^d^	^e^	^f^	^g^	^h^
	**Date**		01/2013	02/2014	08/2015	12/2016	04/2017
**Number of studies**			11	8	21	13	24
**Statistics**	Network model		Bayesian	Bayesian	Bayesian	Bayesian	Frequentist
	Sensitivity analysis		Heterogeneous studies; ethnic group	Ethnic group		Heterogeneous studies; ethnic group	Studies at higher risk of bias^i^
	Covariates		Baseline BCVA and/or CRT	Baseline BCVA and/or CRT		Baseline BCVA and/or CRT	
	Others					Some IPD	
**Participants**	Diabetic macular oedema		√	√	√	√	√
			Significant, focal or diffuse; DME secondary to diabetes involving the centre of the macula; Retinal thickening due to DME/clinically significant macula oedema with DR	Baseline BCVA and CRT varied—24–78 letters	Non-specific	As for Korobelnik 2015	Baseline visual acuity between 20/200 and 20/40; Previously received central/peripheral laser or treatment naïve included
**Interventions**^**k**^	**Aflibercept**		2q4 or 2q8	2 mg; bimonthly	Intravitreal	2q8	2 mg^j^
		+ Laser	√			√	
	**Ranibizumab**		0.5 mg, PRN	0.5 mg, PRN	Intravitreal	0.5 mg PRN or 0.5 mg T&E or 0.3 mg q4	0.5 mg or 0.3 mg
		+ Laser	√	√		√	Deferred
							Prompt
	Dexamethasone (continued)		Implants		Implants		
	Bevacizumab				Intravitreal	1.25 mg	1.25 mg
		+ Laser	√		√	√	
	Triamcinolone acetonide				Intravitreal	4 mg q4/PRN or 4 mg q4	
		+ Laser	√		√	√	
	Pegaptanib						0.3 mg
	Laser		√	√	√		√
		+ Sham injection	√				
	Sham			√			√
**Outcomes**	Binary	ETDRS letters in BCVA	> 10 and > 15 gain; > 10 and > 15 loss	> 10 gain		> 10 and > 15 gain; > 10 and > 15 loss	> 15 gain
		AEs	√		√		√
	Continuous (average change)	In BCVA using ETDRS charts	√		√	√	√
					CMT		CRT measured using OCT
**Conclusions**			Studies of IVT-AFL 2q8 showed improved 12-month visual acuity measures compared with studies of IVR 0.5 mg PRN and dexamethasone 0.7 mg implants based on indirect comparisons	Ranibizumab was non-significantly superior to aflibercept and both anti-VEGF therapies had statistically superior efficacy to laser	Our analysis confirms that intravitreal aflibercept is the most favourable with both BCVA improvement and CMT decrease than other current therapies in the management of DME within 12 months. Vascular endothelial growth factor inhibitors for DME should be used with caution due to systematic AEs. Combined intravitreal triamcinolone with LASER has a stronger efficacy in decreasing CMT than the other interventions in the early stage after injection	This NMA, which incorporated IPD to improve analytic robustness, showed evidence of the superiority of IVT-AFL 2q8 to laser and ranibizumab 0.5 mg PRN. These results were irrespective of adjustment for baseline BCVA	Anti-VEGF drugs given by injection into the eye improve vision in people with diabetic macular oedema as compared to no average improvement with laser photocoagulation. One of these drugs, aflibercept, probably works slightly better after 1 year. There did not appear to be important harms from any of these drugs
**Key results**			There was an increase in the mean best-corrected visual acuity (BCVA) with IVT-AFL 2q8 over IVR 0.5 mg PRN. IVT-AFL 2q8 doubled the proportion of patients gaining ≥ 10 Early Treatment Diabetic Retinopathy Study letters at 12 months compared with dexamethasone 0.7 mg implants. There were no significant differences in safety outcomes between IVT-AFL 2q8 and IVR 0.5 mg PRN or dexamethasone 0.7 mg implants	The efficacy of ranibizumab was numerically, but not statistically, superior to aflibercept. Ranibizumab and aflibercept were statistically superior to laser monotherapy. The probability that ranibizumab is the most efficacious treatment was 73% compared with 14% for aflibercept, 12% for ranibizumab plus laser, and 0% for laser	Intravitreal ranibizumab improved BCVA most significantly in 6 months and intravitreal aflibercept in 12 months. Intravitreal triamcinolone combined with LASER decreased CMT most significantly in 6 months and intravitreal aflibercept in 12 months. Compared with the relatively high rate of ocular AEs in the groups with administration of steroids, systematic AEs occurred more frequently in the groups with vascular endothelial growth factor inhibitors involved	IVT-AFL 2q8 was superior to laser in all analyses. IVT-AFL 2q8 showed strong evidence of superiority (95% credible interval [CrI] did not cross null) versus ranibizumab 0.5 mg PRN for the mean change in BCVA, ≥ 15 ETDRS letter gain, and ≥ 10 ETDRS letter loss. IVT-AFL 2q8 was not superior to ranibizumab 0.5 mg T&E for mean change in BCVA	All three anti-VEGF drugs prevent visual loss and improve vision in people with DMO (high-certainty evidence) People receiving ranibizumab were probably slightly less likely to improve vision compared with aflibercept at 1 year after the start of treatment (moderate-certainty evidence). Approximately three in 10 people improve vision by 3 or more lines with ranibizumab, and one in 10 additional people can achieve this with aflibercept. People receiving ranibizumab and bevacizumab probably have a similar visual outcome at 1 year after the start of treatment (moderate certainty evidence). Aflibercept, ranibizumab, and bevacizumab are similar for common and serious systemic harms (such as any disease leading to hospitalisation, disability, or death) (moderate- or high-certainty evidence) but is less certain for arterial thromboembolic events (mainly stroke, myocardial infarction, and vascular death) and death of any cause (very low-certainty evidence)

*AE* Adverse event, *BCVA* Best-corrected visual acuity, *CI* Credible/confidence interval, *CMT* Central macular thickness, *CRT* Central retinal thickness, *DME* Diabetic macular oedema, *DR* Diabetic retinopathy, *ETDRS* Early Treatment Diabetic Retinopathy Study, *IPD* Individual patient data, *NI* No information, *NMA* Network meta-analysis, *OCT* Optical coherence tomography, *PRN* Pro re nata, *q4* Every 4 weeks, *T&E* Treat-and-extend, *2q8* 2 mg every 8 week

^a^Sorted by search date, empty spaces indicate that the content was not part of the NMA

^b^Including in-process citations and daily update

^c^PubMed

^d^The bibliographies of identified research and review articles. Any abstracts for any unpublished studies at the time of literature review were provided by Bayer HealthCare (Berlin, Germany)

^e^Hand searching of abstracts from ophthalmology congresses (Association for Research in Vision and Ophthalmology [ARVO], American Academy of Ophthalmology [AAO], and European Society of Retina Specialists [EURETINA]), the ClinicalTrials.gov registry, and data on file at Novartis

^f^ClinicalTrials.gov (from January 2015 to December 2016)

^g^The reference lists of published meta-analyses of DME treatment

^h^International Clinical Trials Registry Platform, ISRCTN registry, LILACS; Novartis Clinical Trials database, US National Institutes of Health Ongoing Trials Register ClinicalTrials.gov, and World Health Organization

^i^Post hoc

^j^Regarding drug dose and monitoring/retreatment regimen, Virgili et al. (2018) [17] included schemes that are either on-label or commonly used in clinical practice (such as monthly, bimonthly, PRN, T&E)

^k^No included NMAs included RCTs which investigated conbercept during our search dates (June 2020)

### Methodological quality of the included NMAs

The overall quality of the AMSTAR-2 assessment (Table 2) showed that only the Cochrane NMA [17] was of high quality, and the remaining NMAs had major methodological limitations. The percentage of NMAs that satisfied AMSTAR-2 seven critical domains were as follows: protocol registered before commencement of the review (item 2) ~ 40% (2/5), adequacy of the literature search (item 4) ~ 80% (4/5), justification for excluding individual studies (item 7) ~ 40% (2/5), risk of bias from individual studies being included in the review (item 9) ~ 80% (4/5), appropriateness of meta-analytical methods (item 11) ~ 20% (1/5), consideration of risk of bias when interpreting the results of the review (item 13) ~ 40% (2/5), and assessment of presence and likely impact of publication bias (item 15) ~ 20% (1/5). Regarding the potential sources of conflict of interest (item 16), Zhang et al. [15] and Virgili et al. [17] did not involve authors from the industry, the author teams of Korobelnik et al. [13] and Régnier et al. [14] were from the industry, and Muston et al. [16] were funded by the industry. In addition, funding and conflict of interest information was well reported in all five NMAs. Full details of the assessment and supporting information are provided in Additional file 3.

**Table 2:** Quality assessment by AMSTAR-2 Tool

Items	Korobelnik 2015 [13]	Re´gnier 2014 [14]	Zhang 2016 [15]	Muston 2018 [16]	Virgili 2018 [17]
**Overall assessment**	**Critically low**	**Critically low**	**Critically low**	**Critically low**	**High**
1. Did the research questions and inclusion criteria for the review include the components of PICO? Yes/no	Yes	Yes	No	Yes	Yes
***2. Did the report of the review contain an explicit statement that the review methods were established prior to the conduct of the review and did the report justify any significant deviations from the protocol?*** Yes/partial, yes/no	Partial yes	No	No	No	Partial yes
3. Did the review authors explain their selection of the study designs for inclusion in the review? Yes/no	Yes	No	No	No	No
***4. Did the review authors use a comprehensive literature search strategy?*** Yes/partial, yes/no	Yes	No	Partial yes	Partial yes	Partial yes
5. Did the review authors perform study selection in duplicate? Yes/no	Yes	Yes	No	No	Yes
6. Did the review authors perform data extraction in duplicate? Yes/no	Yes	No	Yes	Yes	Yes
***7. Did the review authors provide a list of excluded studies and justify the exclusions?*** Yes/partial, yes/no	No	Yes	No	No	Yes
8. Did the review authors describe the included studies in adequate detail? Yes/partial, yes/no	Partial yes	Partial yes	Partial yes	Partial yes	Partial yes
***9. Did the review authors use a satisfactory technique for assessing the risk of bias (RoB) in individual studies that were included in the review?*** Yes/partial, yes/no/includes only NRSI/includes only RCTs	Yes	Partial yes	Yes	No	Yes
10. Did the review authors report on the sources of funding for the studies included in the review? Yes/no	No	No	No	No	Yes
***11. If meta-analysis was performed did the review authors use appropriate methods for statistical combination of results?*** Yes/no/no meta-analysis conducted	No	No	No	No	Yes
12. If meta-analysis was performed, did the review authors assess the potential impact of RoB in individual studies on the results of the meta-analysis or other evidence synthesis? Yes/no/no meta-analysis	No	Yes	No	No	Yes
***13. Did the review authors account for RoB in individual studies when interpreting/discussing the results of the review?*** Yes/no	No	Yes	No	No	Yes
14. Did the review authors provide a satisfactory explanation for, and discussion of, any heterogeneity observed in the results of the review? Yes/no	No	No	No	No	Yes
***15. If they performed quantitative synthesis did the review authors carry out an adequate investigation of publication bias (small study bias) and discuss its likely impact on the results of the review?*** Yes/no/no meta-analysis conducted	No	No	No	No	Yes
16. Did the review authors report any potential sources of conflict of interest, including any funding they received for conducting the review? Yes/no	Yes	Yes	Yes	Yes	Yes

AMSTAR: a measurement tool to assess systematic reviews; critical domains in bold italics; rating overall confidence in the results of the review: High (no or one non-critical weakness: the systematic review provides an accurate and comprehensive summary of the results of the available studies that address the question of interest); moderate (more than one non-critical weakness: the systematic review has more than one weakness but no critical flaws. It may provide an accurate summary of the results of the available studies that were included in the review; multiple non-critical weaknesses may diminish confidence in the review, and it may be appropriate to move the overall appraisal down from moderate to low confidence.); low (one critical flaw with or without non-critical weaknesses: the review has a critical flaw and may not provide an accurate and comprehensive summary of the available studies that address the question of interest); critically low (more than one critical flaw with or without non-critical weaknesses: the review has more than one critical flaw and should not be relied on to provide an accurate and comprehensive summary of the available studies)

### Key results of the included NMAs

Best-corrected visual acuity (BCVA) is an important outcome to assess the effect of interventions in people with DME. It is usually measured using Early Treatment Diabetic Retinopathy Study (ETDRS) letters. Different base trials may report this outcome in different ways, and then, in turn, different NMAs can choose different trials and varied ways of reporting. Table 3 shows, how, for the identical binary outcome (the proportion of participants who gain ≥ 10 ETDRS letters at 12 months), different NMAs collected data from different trials, and, partly due to this, arrived at slightly different point estimates—although they all agreed that there was no clear difference between the two treatments (all 95% credible intervals [CrI] straddled ‘one’). Mostly, different decisions for the trial choices according to PICO information are all considered to be clinically meaningful in different NMAs. These differences then lead to results that are not identical. For example, one reviewer may feel that a systematic difference in participants in a particular trial may make it inappropriate to network with data from other trials (e.g. Ishibashi et al. [18], only included in the sensitivity analysis in Régnier et al. [14], included in the main analysis of Korobelnik et al. [13]). Variations in the dosage of treatments may, in the view of one review team, make a study ineligible but be acceptable to other researchers (e.g. Massin et al. [19], included in Régnier et al. [14], excluded from Korobelnik et al. [13]/Muston et al. [16]). Time point of outcome assessment can lead to further differences (e.g. Nguyen et al. [20], included in Régnier et al. [14], excluded from Korobelnik et al. [13]/Muston et al. [16]). These decisions, all made with the best of intentions, lead to the inclusion of different trials contributing to the final—slightly different—results as illustrated in Table 3.

**Table 3:** Summary of three key outcomes on aflibercept versus ranibizumab in included NMAs

	Régnier 2014 [14]	Korobelnik 2015 [13]	Zhang 2016 [15]	Muston 2018 [16]	Virgili 2018 [17]
**Gain ≥ 10 ETDRS letters at 12 months (three NMAs**^**a**^**)**					
**OR** [95% CrI]	**0.63** [0.19 to 1.63]	**1.59** [0.75 to 3.35]	NR	**1.79** [0.63 to 4.06]	NR
**Trials reporting these data**^**b**^** in each NMA**					
Elman 2010 [21] [DRCR.net Protocol I]	Included	Included	NR	Included	NR
Mitchell 2011 [22] [RESTORE]	Included	Included	NR	Included	NR
Korobelnik 2014 [23] [VIVID; VISTA]	Included	Included	NR	Included	NR
Massin 2010 [19] [RESOLVE]	Included	Not included^c^	NR	Not included^c^	NR
Googe 2011 [24] [DRCR.net Protocol J]	Not included^d^	Included	NR	Included	NR
Do DV 2012 [25] [Da VINCI]	Included	Not included^d^	NR	Not included^d^	NR
Ishibashi 2015 [18] [REVEAL]	Not included focus on Asian population^e^	Included^e^	NR	Included	NR
RESPOND [26] [NCT01135914]	Included	Not included^f^	NR	Included	NR
Nguyen 2009 [20] [READ-2]	Included^e^	Not included^g^	NR	Not included^g^	NR
**Average change in BCVA**^**h**^** at 12 months** MD [95% CrI] **(five NMAs)**					
	***4.5**** [1.5 to 7]*^i^	**4.67** [2.45 to 6.87]	**2.07** [− 0.97 to 5.33]	**5.20** [1.90 to 8.52]	**4** [2.5 to 5.5]
**Gain ETDRS letters**^**h**^** at 12 months** OR [95% CrI] **(five NMAs)**					
** ≥ 10**	***0.63*** [0.19–1.63]	**1.59** [0.75–3.35]	NR	**1.79** [0.63–4.06]	NR
** ≥ 15**	NR	NR	NR	**2.30** [1.12–4.20]	**1.33** [1.06–1.67]^j^

*CrI*, credible interval; *BCVA*, best-corrected visual acuity; *ETDRS*, Early Treatment Diabetic Retinopathy Study; *NMA*, network meta-analysis; *NR*, not reported; *OR*, odds ratio

^a^Zhang (2016) and Virgili et al. (2018) did not report this outcome

^b^The additional reasons presented for ‘included’ or ‘not included’ were identified by the author team of this review, not identified in the original texts of NMAs

^c^Data unavailable on ranibizumab 0.5 mg

^d^Unclear reason for exclusion

^e^Included in the sensitivity analysis

^f^Unpublished when NMA conducted

^g^Data only reported at 6 months

^h^Higher values represent better visual acuity measured using ETDRS letters

^i^Data were analysed by the author team of this review (Bayesian network model/random effects, using the ADDIS software), not reported in the original texts of Re´gnier (2014)

^j^Data were risk ratio (RR) and its 95% CrI

In addition, the BCVA measure can be reported as a continuous score of average change (see Table 3—average change in BCVA at 12 months, 95% CrI straddled ‘zero’ indicates no clear differences). We reproduced the results from each NMA and illustrated how the same measure is reported in different ways and different combinations across the five NMAs and found that a pattern does arise. Continuous outcomes all favour aflibercept over ranibizumab. Only Zhang et al. [15] did not reach conventional levels of statistical significance for this outcome (its 95% CrI included a negative value), but all the mean scores do favour the aflibercept group. The binary scores, however, tell a different story—and in no case, when a binary cut-off of ≥ 10 was used—was a statistically significant difference seen (these 95% CrIs straddled ‘one’). In the two later NMAs—including the one considered to be of the highest quality by AMSTAR-2—a binary cut-off of ≥ 15 was employed. For this particular outcome, both NMAs suggest that there exists a statistically significant difference in favour of people allocated to aflibercept (the 95% CrIs were > 1).

In general, it is clear that the reader must continue to think ‘cleanly’ amidst the data which may not be so clean. Below, we discuss some points of particular concern illustrated by these five similar NMAs.

## Discussion

### Network differences

Network meta-analysis is an exciting but still evolving tool employing data from [in these cases] randomised trials in ways by which comparisons of interest can be constructed by using somewhat assumption-heavy observational methods. For example, aflibercept versus ranibizumab is the comparison of interest (referred to as the decision set). Aflibercept may only have been compared with sham injection in randomised trials. Ranibizumab, likewise, may only have been compared with sham injection. We use the term ‘supplementary set’ to refer to interventions, such as sham injection, that are included in the network meta-analysis for the purpose of improving inference among interventions in the decision set [4]. As different selections for supplementary set, different network structures will be conducted for the same clinical problem. For example, aflibercept may have been compared with sham injection in some trials and laser in others. Ranibizumab also may have some sham-controlled trials and some where it has competed against laser. One review may choose the sham-controlled trials as the supplementary set, and another review may choose the laser-controlled trials. In selecting which competing interventions to include in the decision set, researchers should ensure that the transitivity assumption is likely to hold [4, 27]. The choice of supplementary set is mostly based on clinical considerations [4]. Including more interventions in the network may provide more information, which leads to more precise results. Meanwhile, it brings the risk of employment of non-valid indirect comparisons and of varied and ill-understood assumptions. When theoretical assumptions are guaranteed (transitivity and consistency), there is no absolute right or wrong in the construction of a network structure. The reader of the review should carefully consider if she/he feels the network indirect comparisons are sensible and making best use of available data.

### PICO differences

From within even a few trials, the multiple choices of what data to select begin to become obvious. Of course, these choices should be based on sound clinical rationale and taken when as blind as possible to the base trials and their results—hence the value of a peer-reviewed protocol for the conduct of the trial. It should not be a surprise that clinicians and researchers evolve their ideas and differ even at the same time. Scientific questions merit replication and, powerful and reassuring in this illustration, is that although the point estimates differ across the trials, the findings are, for the outcomes in Table 3, essentially the same—no matter what way the data are used. Readers need to consider and understand what participants are included—and excluded—in the trial; what treatments are its focus and if there are omissions; and what outcomes are being reported and why those choices were taken. On the other hand, some studies [28, 29] have shown that while some independent replication of meta-analyses (or overviews of reviews [30]) by different teams may be useful, there is considerable overlap and potential redundancy in published NMAs. Replication can add value or be wasteful, depending on the topic and quality of research.

### Different data from the same measures of effect

Continuous data from a scale or measure provide more detailed information than dichotomous data from the same scale or measure. The dichotomous or binary is often a crude and even arbitrary cut-off within an ostensibly continuous measure. Continuous measures are, however, often a research fabrication and not truly continuous. In the real world, a 10-point decline in a score from 90 to 80 may have quite a different meaning than a 10-point decline on the same measure from 20 to 10. In addition, clinicians and patients first tend to seek if the treatment will help them, for example, ‘get better’ (a question that merits a binary answer) and then, only as second preference, seek information on the degree of improvement (meriting an answer on a continuous scale). In the examples used in this paper, there is reasonable consistency that aflibercept does help shift the mean scores in a positive direction (continuous) when compared with ranibizumab. Crude though it may be, however, the binary is easier to interpret clinically and, if great enough is likely to mean ‘getting better’ or ‘making a substantial difference’ despite shortcomings of the so-called continuous measure. In the examples in Table 3, the average change improvements seem relatively consistently to be a matter of around 4 points. It is problematic to really understand what this may mean for any one patient’s life. In averaging across the groups, something may be lost, however, that is revealed in the binary, and Table 3 gives good evidence for speculation. What trials that report a ≥ 10-point gain consistently are reviewed to show no clear difference between aflibercept and ranibizumab, but the two latest trials have a new binary to report (≥ 15-point gain) and both show an advantage for those allocated to aflibercept. Perhaps in the averaging across all people in the trials, there has been a masking of an important group of people who respond better to aflibercept. But these are clinical and research points of debate. Overall, the five NMAs have reported results that are complicated, thought-provoking, but not truly inconsistent with each other. The reader needs to consider the value of the outcome for their need. The researchers may favour the continuous measure of function, the clinician or patient, the binary cut-off for better/not better, and the policy maker the economics.

### Differences in what is truly significant

As for individual studies, results for meta-analyses are reported with a point estimate with an associated confidence interval. The confidence interval describes the uncertainty inherent in any estimate and describes a range of values within which we can be reasonably confident that the true effect lies [4]. If the confidence interval is relatively narrow (e.g. 2.5 to 5.5 in Virgili et al. [17], Table 3), the effect size on the mean change in BCVA is known reasonably precisely. If the interval is wider (e.g. 1.5 to 7 in Régnier et al. [14], Table 3), the uncertainty is greater, although there may still be enough precision to make decisions about the utility of the finding. Intervals that are very wide indicate that we have little knowledge about where the true effect actually is and our certainty is eroded.

When the synthesis of continuous data produces confidence intervals of the data’s point estimates that are both on the positive side (e.g. Muston et al.’s [16] 1.90 to 8.52, Table 3), or both on the negative, then the finding has reached a pre-stated level of statistical significance. When the synthesis of binary data produces confidence intervals of the data’s point estimates that are both greater than one (e.g. Muston et al.’s [16] 1.12–4.20, Table 3), or both less than one, then the finding has reached a pre-stated level of statistical significance. However, as has been suggested above, the statistically significant findings of an outcome measure may not have a great clinical impact. An outcome may be statistically significant, yet clinically insignificant. It is easy for confusion to arise when the same data are commented upon by one set of reviewers discussing findings from the statistical perspective and another set of reviewers considering the clinical meaning of the data. Careful consideration is required from the reader to understand the assessment of the reviewers—are they reporting the clinical or statistical perspective—or a mixture of both.

A further danger of differing interpretations of the same findings lies in when confidence intervals straddle 0 (for continuous data) or 1 (for binary data—as for all the ≥ 10-point gain findings in Table 3). The findings are certainly not statistically significant, but it is easy for reviewers and readers of the trials to confuse ‘no evidence of an effect’ with ‘evidence of no effect’. When confidence intervals are wide, for example, the 0.63 to 4.06 of Muston et al. [16] in Table 3, they straddle 1 or unity. In this case, it is wrong to claim that aflibercept has ‘no effect’ or is ‘no different’ from ranibizumab—both statements carry too much certainty. As the confidence interval for the estimate of the difference in the effects of the treatments overlaps with no effect (in this binary example, 1), the analysis is compatible with both a true beneficial effect and a true harmful effect. It is true that there is no clear difference, but one drug is not clearly different from the other. If a true beneficial effect is mentioned in the conclusion, a true harmful effect should also be mentioned and discussed. It is so easy for reviewers and readers of those trials to take one side of a message from the same data and leave another half of the message less considered. Again, the reader has to be vigilant that balance has been achieved by the reviewers and if it has not, then be balanced themselves.

As always, really thinking about the meaning of findings is key. Together, the point estimate and confidence interval provide information to assess the effects of the intervention on the outcome. For example, in the evaluation of these drugs on BCVA, it could have been decided that it would be clinically useful if the medication increased BCVA from baseline by 5 letters—and at the very least 2 letters. Virgili et al. [17] report an effect estimate of an increase from a baseline of four letters with a 95% confidence interval from 2.5 to 5.5 letters. If this finding is based on good methods (see above), this allows the conclusion that aflibercept was useful since both the point estimate and the entire range of the interval exceed the criterion of an increase of two letters. The Régnier et al. [14] trial reported a similar point estimate (4.5 letters) but with a wider interval from 1.5 to 7 letters. In this case, although it could still be concluded that the best estimate of the aflibercept effect is that it provides net benefit, the reader could not be so confident as the possibility still has to be entertained that the effect could be between 1.5 and 2 letters—a low range that had been pre-specified to be of little clinical value. So, in this example, the latter, higher-quality trial had the confidence intervals that were reassuring of a net benefit for one compound. But the contrast of Régnier et al. [14] and Virgili et al. [17] serves well to illustrate how very similar findings may justify subtly different implications. The reviewers carry a responsibility to help the reader through clear reporting and thoughtful inclusive explanations—but where this has not happened, the readers may have to do this for themselves.

## Conclusions

We have summarised the methods and findings of five NMAs of the same topic which produced what seemed like somewhat different findings from similar data sets. Closer inspection of each of these trials shows how the methods, including the searches and analyses, all differ, but the findings, although presented differently and sometimes interpreted differently, were similar. That five different NMAs—all using different datasets, networks, and review teams broadly producing similar findings—must be reassuring that there is some consistency in the results of the aflibercept/ranibizumab comparison.

As always, the critical reader of a review should think about the review in detail. This is helped by long-established checklists [27]. Furthermore, Grading of Recommendations Assessment, Development, and Evaluation (GRADE) offers a transparent and structured process for developing and presenting summaries of evidence, including its quality, for systematic reviews and recommendations in health care [31].

As is common in different trials and reviews, outcomes—even the same measures—can be legitimately reported in several different ways. There is no avoiding the need to think through what the numbers really mean in terms of people, services, and policies. This may necessitate careful, subtle, humane, and expert consideration.

### Supplementary Information

**Additional file 1. **Search strategies.

**Additional file 2. **Details of included studies in these NMAs.

**Additional file 3. **Quality assessment by AMSTAR-2 Tool (Support information in detail).

## Data Availability

The datasets used and/or analysed during the current study are available from the corresponding author upon reasonable request.

## Abbreviations

BCVA: Best-corrected visual acuity
ETDRS: Early Treatment Diabetic Retinopathy Study
DME: Diabetic macular oedema
DR: Diabetic retinopathy
GRADE: Grading of Recommendations Assessment, Development, and Evaluation
NMA: Network meta-analyses
PICO: Participants, interventions, comparators, outcomes
PRN: Pro re nata
QoL: Quality of life
T&E: Treat and extend
VEGF: Vascular endothelial growth factor
