# Supplementary material for: Diversity when interpreting evidence in network meta-analyses (NMAs) on similar topics: an example case of NMAs on diabetic macular oedema
Source: Syst Rev. 2023 Oct 7;12:189. doi: 10.1186/s13643-023-02349-4 (PMC10559427; doi:10.1186/s13643-023-02349-4)
Supplement: Supplementary file 1 — Additional file 1. Search strategies. [file 13643_2023_2349_MOESM1_ESM.docx]

**Search strategies**

*PubMed*

1. ("diabetic macular edema"[tw] OR "diabetic macula edema"[tw] OR "DME" OR (("Macular Edema"[Mesh] OR "macular edema"[tw] OR "macular edemas"[tw] OR "macula edema"[tw] OR "macula edemas"[tw] OR "macular oedema"[tw] OR "macular oedemas"[tw] OR "macula oedema"[tw] OR "macula oedemas"[tw]) AND ("Diabetes Mellitus"[mesh] OR diabet*[tw])))
2. ("aflibercept"[tw] OR "ranibizumab"[tw] OR "conbercept"[tw])
3. ("systematic"[sb] OR "Systematic Review"[Publication Type] OR "Systematic Reviews as Topic"[Mesh] OR "Systematic Review"[tw] OR "Meta-Analysis"[Publication Type] OR "Meta-Analysis as Topic"[Mesh] OR "Network Meta-Analysis"[Mesh] OR "Meta-analysis"[tw] OR "Metaanalysis"[tw] OR Meta-analy*[tw] OR Metaanaly*[tw])
4. 1 AND 2 AND 3

*EMBASE*

1. ("diabetic macular edema"/ OR "diabetic macular edema".mp OR "diabetic macula edema".mp OR "DME" OR ((exp "Macular Edema"/ OR "macular edema".mp OR "macular edemas".mp OR "macula edema".mp OR "macula edemas".mp OR "macular oedema".mp OR "macular oedemas".mp OR "macula oedema".mp OR "macula oedemas".mp) AND (exp "Diabetes Mellitus"/ OR diabet*.mp)))
2. ("aflibercept".mp OR "ranibizumab".mp OR "conbercept".mp)
3. ("Systematic Review"/ OR "Systematic Review".mp OR exp "Meta Analysis"/ OR "Meta-analysis".mp OR "Metaanalysis".mp OR "Meta-analy*".mp OR Metaanaly*.mp)
4. 1 AND 2 AND 3

*Cochrane Library*

1. (("diabetic macular edema" OR "diabetic macula edema" OR "DME" OR (("Macular Edema" OR "macular edema" OR "macular edemas" OR "macula edema" OR "macula edemas" OR "macular oedema" OR "macular oedemas" OR "macula oedema" OR "macula oedemas") AND ("Diabetes Mellitus" OR diabet*))) AND ("aflibercept" OR "ranibizumab" OR "conbercept")):ti,ab,kw
2. ("aflibercept" OR "ranibizumab" OR "conbercept"):ti,ab,kw
3. 1 AND 2

*Web of Science*

1. TS=(("diabetic macular edema" OR "diabetic macula edema" OR "DME" OR (("Macular Edema" OR "macular edema" OR "macular edemas" OR "macula edema" OR "macula edemas" OR "macular oedema" OR "macular oedemas" OR "macula oedema" OR "macula oedemas") AND ("Diabetes Mellitus" OR diabet*))) AND ("aflibercept" OR "ranibizumab" OR "conbercept"))
2. TS=("Systematic Review" OR "Meta Analysis" OR "Metaanalysis" OR Metaanaly* OR "meta-analy*")
3. 1 AND 2

*Cnki*

(SU %=糖尿病性黄斑水肿+糖尿病黄斑水肿+DME*黄斑+糖尿病*黄斑水肿 OR TKA='糖尿病 /NEAR 2 黄斑水肿'+DME*黄斑) AND (SU%=Avastin+Bevacizumab+Ranibizumab+Lucentis+Aflibercept+conbercept+安维汀+贝伐珠单抗+贝伐单抗+阿瓦斯汀+阿瓦斯丁+兰尼单抗+雷珠单抗+诺适得+康博西普+康柏西普+阿柏西普+阿普西柏+采视明+VEGF单抗+抗VEGF+血管内皮生长因子单抗+抗血管内皮生长因子 OR TKA=Avastin+Bevacizumab+Ranibizumab+Lucentis+Aflibercept+conbercept+安维汀+贝伐珠单抗+贝伐单抗+阿瓦斯汀+阿瓦斯丁+兰尼单抗+雷珠单抗+诺适得+康博西普+康柏西普+阿柏西普+阿普西柏+采视明+VEGF单抗+抗VEGF+血管内皮生长因子单抗+抗血管内皮生长因子) AND (SU%=META+荟萃+系统综述+系统评价+评价综述 OR TKA=META+荟萃+系统综述+系统评价+评价综述)

*Wanfang*

主题:(DME*黄斑+糖尿病*黄斑水肿)*(Avastin+Bevacizumab+Ranibizumab+Lucentis+Aflibercept+conbercept+"安维汀"+"贝伐珠单抗"+"贝伐单抗"+"阿瓦斯汀"+"阿瓦斯丁"+"兰尼单抗"+"雷珠单抗"+"诺适得"+"康博西普"+"康柏西普"+"阿柏西普"+"阿普西柏"+"采视明"+"VEGF单抗"+"抗VEGF"+"血管内皮生长因子单抗"+"抗血管内皮生长因子")*(META+荟萃+"系统综述"+"系统评价"+"评价综述")

*VIP*

(M=糖尿病黄斑水肿+糖尿病性黄斑水肿+DME*黄斑+糖尿病*黄斑水肿 OR R=糖尿病黄斑水肿+糖尿病性黄斑水肿+DME*黄斑+糖尿病*黄斑水肿) AND (M=Avastin+Bevacizumab+Ranibizumab+Lucentis+Aflibercept+conbercept+安维汀+贝伐珠单抗+贝伐单抗+阿瓦斯汀+阿瓦斯丁+兰尼单抗+雷珠单抗+诺适得+康博西普+康柏西普+阿柏西普+阿普西柏+采视明+VEGF单抗+抗VEGF+血管内皮生长因子单抗+抗血管内皮生长因子 OR R=Avastin+Bevacizumab+Ranibizumab+Lucentis+Aflibercept+conbercept+安维汀+贝伐珠单抗+贝伐单抗+阿瓦斯汀+阿瓦斯丁+兰尼单抗+雷珠单抗+诺适得+康博西普+康柏西普+阿柏西普+阿普西柏+采视明+VEGF单抗+抗VEGF+血管内皮生长因子单抗+抗血管内皮生长因子) AND (M=META+荟萃+系统综述+系统评价+评价综述 OR R=META+荟萃+系统综述+系统评价+评价综述)
