# Supplementary material for: Diversity when interpreting evidence in network meta-analyses (NMAs) on similar topics: an example case of NMAs on diabetic macular oedema
Source: Syst Rev. 2023 Oct 7;12:189. doi: 10.1186/s13643-023-02349-4 (PMC10559427; doi:10.1186/s13643-023-02349-4)
Supplement: Supplementary file 2 — Additional file 2. Details of included studies in these NMAs. [file 13643_2023_2349_MOESM2_ESM.docx]

**Details of included studies in these NMAs**

|  | | | | | Study ID^a^ | | | | |
| --- | --- | --- | --- | --- | --- | --- | --- | --- | --- |
|  | | | | | **Korobelnik**  **2015** | **Régnier 2014** | **Zhang 2016** | **Muston 2018** | **Virgili**  **2018** |
| **Search** | | Cochrane | | | ✓ | ✓ | ✓ | ✓ | ✓ |
|  |  | EMBASE | | | ✓ | ✓ | ✓ | ✓ | ✓ |
|  |  | MEDLINE | | | ✓^b^ | ✓^b^ | ✓^c^ | ✓^b^ | ✓ |
|  |  | Others | | | ✓^d^ | ✓^e^ | ✓^f^ | ✓^g^ | ✓^h^ |
|  |  | **Date** | | | 01/2013 | 02/2014 | 08/2015 | 12/2016 | 04/2017 |
| **Number of studies** | | | | | 11 | 8 | 21 | 13 | 24 |
| **Key inclusion criteria** | | | | | - RCT; - Patients with DME; - Eylea/VEGF Trap-Eye/aflibercept; Anti-VEGF treatments (any including ranibizumab/Lucentis, bevacizumab/Avastin, and pegaptanib/Macugen); Intravitreal steroids (any including triamcinolone, Subtenon injections fluocinolone acetonide/Iluvien, dexamethasone/ Ozurdex, and implants); Laser treatments; Placebo, best standard care, masked control, sham, and eye drops. | - RCT; - Patients with DME; - At least two comparators of interest (sham injections plus rescue laser, ranibizumab 0.5 mg pro re nata [as needed], ranibizumab 0.5 mg pro re nata plus laser, aflibercept 2.0 mg bi-monthly [every 2 months] and prompt laser photocoagulation therapy); - Studies published in English, French and German; - The outcome of interest had to be measured at 6 or 12 months from study baseline, with 12 month data used for the analysis where available. | - RCT; - Patients with DME (any type of diabetes); - follow-up of more than six months; - efficacy outcomes including BCVA and CMT; - only the articles containing complete data were included. | - As for Korobelnik 2015 - Only trials that reported the randomised controlled results at 12 months for mean change from baseline in BCVA (ETDRS letters) (continuous outcome), and the proportion of patients achieving gain/loss of ≥10 and ≥ 15 ETDRS letters (binary outcomes) were included; - Treatment regimens of interest for European practice were IVT-AFL 2 mg every 8 weeks (2q8) after 5 initial doses, ranibizumab 0.5 mg PRN, ranibizumab 0.5 mg T&E, and laser photocoagulation. | - RCT; - Patients wiwith DME for whom anti-VEGF treatment is indicated; - Any antiangiogenic drug with anti-VEGF modalities compared with another drug with anti-VEGF modalities, laser treatment, sham treatment or no treatment; - Regarding drug dose and monitoring/retreatment regimen, in efficacy analyses we included schemes that are either on-label or commonly used in clinical practice, such as the PRN regimen; - Particularly, both 0.3 mg and 0.5 mg ranibizumab dose are included as available in studies. These two ranibizumab doses were merged into one group in our NMA since studies suggest no difference between them when used monthly; - Regarding aflibercept, we selected the bi-monthly retreatment regimen since this is the approved label in the USA. |
| **Key exclusion criteria** | | | | | - Studies of less than 3 months follow-up; - Systemic treatments (alone or in combination with intervention); Surgery (alone or in combination with intervention); Subtenon injections; - Studies that were connected by one arm only and did not form a closed network, unless they included comparators of interest; Studies that formed loops but did not lie along the path between IVT-AFL 2q8 versus comparators of interest (IVR 0.5 mg PRN, or implants of dexamethasone 0.7 mg or fluocinolone acetonide 0.2 μg/day); Studies that did not report 12-month outcomes. | - Studies with single treatment arms; - RCTs that not report the outcome patients achieving a gain in BCVA of at least 10 letters (2 lines) on the ETDRS scale; - Studies focusing on a specific ethnic group were not included in the base-case analysis but were included in the sensitivity analyses. | None | - Because it is not licensed for ophthalmic use, bevacizumab was not included in the analysis. | - We remark that steroids may be compared with anti-VEGF drugs but this needs a different approach, specifically patient subgroups and timing, and their inclusion could lead to violation of similarity in a review aiming to compare different anti-VEGF drugs such as this. |
| **Participants** | Diabetic macular edema | | | | ✓ | ✓ | ✓ | ✓ | ✓ |
|  |  |  |  |  | Significant, focal or diffuse | Baseline BCVA & CRT varied - 24-78 letters |  | As for Korobelnik 2015 | Baseline visual acuity between 20/200 and 20/40 |
|  |  |  |  |  | DME secondary to diabetes involving the center of the macula |  |  |  |  |
|  |  |  |  |  | retinal thickening due to DME/clinically significant macula edema with DR |  |  |  | previously received central/peripheral laser or treatment naïve included |
| **Interventions** | **aflibercept** | | |  | 2q4 or 2q8 | 2 mg; bimonthly | intravitreal | 2q8 | 2 mg |
|  |  |  |  | + laser | ✓ |  |  | ✓ |  |
|  | **ranibizumab** | | |  | 0.5 mg, PRN | 0.5 mg, PRN | intravitreal | 0.5 mg, PRN or 0.5 mg T&E or 0.3 mg, q4 | 0.5 mg or 0.3 mg |
|  |  |  |  | + laser | ✓ | ✓ |  | ✓ | deferred |
|  |  |  |  |  |  |  |  |  | prompt |
|  | dexamethasone | | | | implants |  | implants |  |  |
|  | bevacizumab | | |  |  |  | intravitreal | 1.25 mg | 1.25 mg |
|  |  |  |  | + laser | ✓ |  | ✓ | ✓ |  |
|  | triamcinolone acetonide | | |  |  |  | intravitreal | 4 mg, q4/PRN or 4 mg, q4 |  |
|  |  |  |  | + laser | ✓ |  | ✓ | ✓ |  |
|  | pegaptanib | | |  |  |  |  |  | 0.3 mg |
|  | Laser | |  | | ✓ | ✓ | ✓ |  | ✓ |
|  |  |  | + sham injection | | ✓ |  |  |  |  |
|  | Sham | | | |  | ✓ |  |  | ✓ |
| **Included studies in detail** (the number of NMAs included the displayed study) | | | | |  |  |  |  |  |
| Ahmadieh2008 (2) | | | | |  |  | ✓ |  | ✓ |
| Audren2006 (1) | | | | |  |  | ✓ |  |  |
| ARVOE (1) | | | | |  |  | ✓ |  |  |
| Arevalo2013 (1) | | | | |  |  | ✓ |  |  |
| Azad2012 (1) | | | | |  |  |  |  | ✓ |
| Berger2015 (1) | | | | |  |  | ✓ |  |  |
| BOLT (2) | | | | |  |  | ✓ |  | ✓ |
| DA VINCI (2) | | | | |  | ✓ |  |  | ✓ |
| DRCR.net Protocol I (5) | | | | | ✓ | ✓ | ✓ | ✓ | ✓ |
| DRCR.net Protocol J (2) | | | | | ✓ |  |  | ✓ |  |
| DRCR.net Protocol T (3) | | | | |  |  | ✓ | ✓ | ✓ |
| Ekinci2014 (1) | | | | |  |  |  |  | ✓ |
| IBETA Abstract (1) | | | | | ✓ |  |  |  |  |
| Gillies2014 (1) | | | | |  |  | ✓ |  |  |
| Lam2007 (1) | | | | |  |  | ✓ |  |  |
| Lopez-Galvez2014 (1) | | | | |  |  |  |  | ✓ |
| LUCIDATE (4) | | | | | ✓ |  | ✓ | ✓ | ✓ |
| Macugen2005 (1) | | | | |  |  |  |  | ✓ |
| Macugen 2011 (1) | | | | |  |  |  |  | ✓ |
| Maia et al (1) | | | | | ✓ |  |  |  |  |
| Nepomuceno2013 (2) | | | | |  |  | ✓ |  | ✓ |
| PLACID (2) | | | | | ✓ |  | ✓ |  |  |
| READ-2 (3) | | | | |  | ✓ | ✓ |  | ✓ |
| RELATION (2) | | | | | ✓ |  |  |  | ✓ |
| RESOLVE (3) | | | | |  | ✓ | ✓ |  | ✓ |
| RESPOND (4) | | | | |  | ✓ | ✓ | ✓ | ✓ |
| RESTORE (5) | | | | | ✓ | ✓ | ✓ | ✓ | ✓ |
| RETAIN (1) | | | | |  |  |  | ✓ |  |
| REVEAL (4) | | | | | ✓ |  | ✓ | ✓ | ✓ |
| RIDE (2) | | | | |  |  |  | ✓ | ✓ |
| RISE (2) | | | | |  |  |  | ✓ | ✓ |
| Soheilian2012 (2) | | | | |  |  | ✓ |  | ✓ |
| Shoeibi2013 (1) | | | | |  |  | ✓ |  |  |
| Synek2011 (1) | | | | |  |  | ✓ |  |  |
| Turkoglu2015 (1) | | | | |  |  |  |  | ✓ |
| VISTA-DME (4) | | | | | ✓ | ✓ |  | ✓ | ✓ |
| VIVID-DME (4) | | | | | ✓ | ✓ |  | ✓ | ✓ |
| VIVID-EAST (1) | | | | |  |  |  | ✓ |  |
| Wiley2016 (1) | | | | |  |  |  |  | ✓ |

a Sorted by search date; b Including In-Process Citations and Daily Update; c PubMed; d the bibliographies of identified research and review articles; Any abstracts for any unpublished studies at the time of literature review were provided by Bayer HealthCare (Berlin, Germany); e hand searching of abstracts from ophthalmology congresses (Association for Research in Vision and Ophthalmology [ARVO], American Academy of Ophthalmology [AAO] and European Society of Retina Specialists [EURETINA]), the ClinicalTrials.gov registry, and data on file at Novartis.; f ClinicalTrials.gov (from January 2015 to December 2016); g the reference lists of published meta-analyses of DME treatment; h International Clinical Trials Registry Platform; ISRCTN registry; LILACS; Novartis Clinical Trials database; US National Institutes of Health Ongoing Trials Register ClinicalTrials.gov; World Health Organization.
References for “***Included studies in detail***”:

| Ahmadieh2008 | | Ahmadieh H, Ramezani A, Shoeibi N, Bijanzadeh B, Tabatabaei A, Azarmina M, et al. Intravitreal bevacizumab with or without triamcinolone for refractory diabetic macular edema; a placebo-controlled, randomized clinical trial. Graefe's archive for clinical and experimental ophthalmology = Albrecht von Graefes Archiv fur klinische und experimentelle Ophthalmologie. 2008 Apr; 246(4):483–9. PMID: 17917738 |
| --- | --- | --- |
| Audren2006 | Audren F, Erginay A, Haouchine B, Benosman R, Conrath J, Bergmann JF, et al. Intravitreal triamcinolone acetonide for diffuse diabetic macular oedema: 6-month results of a prospective controlled trial. Acta ophthalmologica Scandinavica. 2006 Oct; 84(5):624–30. PMID: 16965492 | |
| ARVOE | Pappas GD, Adam CI, Papageorgioy E, Kefalogiannis N, Fanouriakis H. Triamcinolone and Grid Laser versus Bevacizumab Alone for the Treatment of Diabetic Macular Edema. Iovs [Internet]. 2008:[ARVO E- abstract 3483 pp.]. Available from: http://onlinelibrary.wiley.com/o/cochrane/clcentral/articles/124/CN-00746124/frame.html. | |
| Arevalo2013 | Arevalo JF, Lasave AF, Wu L, Diaz-Llopis M, Gallego-Pinazo R, Alezzandrini AA, et al. Intravitreal bevacizumab plus grid laser photocoagulation or intravitreal bevacizumab or grid laser photocoagulation for diffuse diabetic macular edema: results of the Pan-american Collaborative Retina Study Group at 24 months. Retina (Philadelphia, Pa). 2013 Feb; 33(2):403–13. | |
| Azad2012 | AzadR, SainS, SharmaYR, MahajanD. Comparison of intravitreal bevacizumab, intravitreal triamcinolone acetonide, and macular grid augmentation in refractory diLuse diabetic macular edema: A prospective, randomized study. Oman Journal of Ophthalmology 2012; Vol. 5, issue 3:166-70. | |
| Berger2015 | Berger A, Sheidow T, Cruess AF, Arbour JD, Courseau AS, De Takacsy F. Efficacy/safety of ranibizumab monotherapy or with laser versus laser monotherpay in DME. Canadian Journal of Ophthalmology. 2015 Jun; 50(3):209–16. doi: 10.1016/j.jcjo.2014.12.014 PMID: 26040221 | |
| BOLT | Michaelides M, Kaines A, Hamilton RD, Fraser-Bell S, Rajendram R, Quhill F, et al. A prospective randomized trial of intravitreal bevacizumab or laser therapy in the management of diabetic macular edema (BOLT study) 12-month data: report 2. Ophthalmology. 2010 Jun; 117(6):1078–1086.e2. doi: 10.1016/j.ophtha.2010.03.045 PMID: 20416952 | |
| DA VINCI | Do DV, Nguyen QD, Boyer D, Schmidt-Erfurth U, Brown DM, et al. (2012) One-year outcomes of the da Vinci Study of VEGF Trap-Eye in eyes with diabetic macular edema. Ophthalmology 119: 1658–1665. | |
| DRCR.net Protocol I | Elman MJ, Aiello LP, Beck RW, Bressler NM, Bressler SB, Edwards AR, et al. Randomized trial evaluating ranibizumab plus prompt or deferred laser or triamcinolone plus prompt laser for diabetic macular edema. Ophthalmology. 2010;117:1064–77. | |
| DRCR.net Protocol J | Googe J, Brucker AJ, Bressler NM, Qin H, Aiello LP, Antoszyk A, et al. Randomized trial evaluating short-term effects of intravitreal ranibizumab or triamcinolone acetonide on macular edema after focal/grid laser for diabetic macular edema in eyes also receiving panretinal photocoagulation. Retina. 2011;31:1009–27. | |
| DRCR.net Protocol T | Diabetic Retinopathy Clinical Research Network, Wells JA, Glassman AR, et al. Aflibercept, bevacizumab, or ranibizumab for diabetic macular edema. N Engl J Med. 2015;372:1193–203. | |
| Ekinci2014 | EkinciM, CeylanE, CakiciO, TanyildizB, OlcaysuO, CagatayHH. Treatment of macular edema in diabetic retinopathy: Comparison of the eLicacy of intravitreal bevacizumab and ranibizumab injections. Expert Review of Ophthalmology 2014;9(2):139-43. | |
| IBETA Abstract | Almeida FP, Katayama BY, Messias A, Fisher M, Paccola ML, Costa RA, et al. Macular laser photocoagulation combined with intravitreal bevacizumab or triamcinolone for diabetic macular edema. Abstract 1300. Presented at: 2011 Annual Meeting of the Association for Research in Vision and Ophthalmology (ARVO), May 1–5, 2011; Fort Lauderdale, FL. | |
| Gillies2014 | Gillies MC, Lim LL, Campain A, Quin GJ, Salem W, Li J, et al. A randomized clinical trial of intravitreal bevacizumab versus intravitreal dexamethasone for diabetic macular edema: the BEVORDEX study. Ophthalmology. 2014 Dec; 121(12):2473–81. doi: 10.1016/j.ophtha.2014.07.002 PMID: 25155371 | |
| Lam2007 | Lam DS, Chan CK, Mohamed S, Lai TY, Lee VY, Liu DT, et al. Intravitreal triamcinolone plus sequential grid laser versus triamcinolone or laser alone for treating diabetic macular edema: six-month outcomes. Ophthalmology. 2007 Dec; 114(12):2162–7. PMID: 17459479 | |
| Lopez-Galvez2014 | Lopez-GalvezMI, AriasL, RouraM. ELicacy and safety profile of ranibizumab versus laser photocoagulation in patients with diabetic macular edema. Re-Des Study. Ophthalmologica 2014;232:115. | |
| LUCIDATE | Comyn O, Sivaprasad S, Peto T, Neveu MM, Holder GE, Xing W, et al. A randomized trial to assess functional and structural effects of ranibizumab versus laser in diabetic macular edema (the LUCIDATE study). American journal of ophthalmology. 2014 May; 157(5):960–70. doi: 10.1016/j.ajo.2014.02.019 PMID: 24531025 | |
| Macugen2005 | Cunningham ET Jr, Adamis AP, Altaweel M, Aiello LP, Bressler NM, D'Amico DJ, et al. A phase II randomized doublemasked trial of pegaptanib, an anti-vascular endothelial growth factor aptamer, for diabetic macular edema. Ophthalmology 2005;112(10):1747-57. | |
| Macugen 2011 | Sultan MB, Zhou D, LoEus J, Dombi T, Ice KS, Macugen 1013 Study Group. A phase 2/3, multicenter, randomized, doublemasked, 2-year trial of pegaptanib sodium for the treatment of diabetic macular edema. Ophthalmology 2011;118(6):1107-18. | |
| Maia et al | Maia OO Jr, Takahashi BS, Costa RA, Scott IU, Takahashi WY. Combined laser and intravitreal triamcinolone for proliferative diabetic retinopathy and macular edema: one-year results of a randomized clinical trial. Am J Ophthalmol. 2009;147:291–7. | |
| Nepomuceno2013 | Nepomuceno AB, Takaki E, Paes de Almeida FP, Peroni R, Cardillo JA, Siqueira RC, et al. A prospective randomized trial of intravitreal bevacizumab versus ranibizumab for the management of diabetic macular edema. American Journal of Ophthalmology 2013;156(3):502-10. | |
| PLACID | Callanan DG, Gupta S, Boyer DS, Ciulla TA, Singer MA, Kuppermann BD, et al. Dexamethasone intravitreal implant in combination with laser photocoagulation for the treatment of diffuse diabetic macular edema. Ophthalmology. 2013;120:1843–51. | |
| READ-2 | Nguyen QD, Shah SM, Heier JS, Do DV, Lim J, et al. (2009) Primary end point (six months) results of the Ranibizumab for Edema of the mAcula in diabetes (READ-2) study. Ophthalmology 116: 2175–2181 e2171. | |
| RELATION | Ristau T, Voegeler J, Lang G, Liakopoulos S, RELATION Study Group. Relevance of inner versus outer retinal thickness in diabetic macular edema in the RELATION study. Abstract 2376. Presented at: 2013 Annual Meeting of the Association for Research in Vision and Ophthalmology (ARVO), May 5–9, 2013; Seattle, WA.  Lohmann C, Voegeler J, Liakopoulos S, Wiedemann P, Spital G, Lang G, et al. Double-masked trial demonstrates superiority of combined ranibizumab plus laser versus laser in patients with diabetic macular edema with or without proliferative diabetic retinopathy. Abstract 1239. Presented at: 2013 Annual Meeting of the Association for Research in Vision and Ophthalmology (ARVO), May 5–9, 2013; Seattle, WA. | |
| RESOLVE | Massin P, Bandello F, Garweg JG, Hansen LL, Harding SP, et al. (2010) Safety and efficacy of ranibizumab in diabetic macular edema (RESOLVE Study): a 12-month, randomized, controlled, double-masked, multicenter phase II study. Diabetes Care 33: 2399–2405. | |
| RESPOND | RESPOND: Safety, Efficacy and Cost-efficacy of Ranibizumab (Monotherapy or Combination With Laser) in the Treatment of Diabetic Macular Edema (DME). Novartis data on file to be submitted for publication | |
| RESTORE | Mitchell P, Bandello F, Schmidt-Erfurth U, Lang GE, Massin P, Schlingemann RO, et al. The RESTORE study: ranibizumab monotherapy or combined with laser versus laser monotherapy for diabetic macular edema. Ophthalmology. 2011;118:615–25. | |
| RETAIN | Prunte C, Fajnkuchen F, Mahmood S, et al. Ranibizumab 0.5 mg treat-andextend regimen for diabetic macular oedema: the RETAIN study. Br J Ophthalmol. 2016;100:787–95. | |
| REVEAL | Ishibashi T, Li X, Koh A, Lai TY, Lee FL, Lee WK, et al. The REVEAL Study: Ranibizumab Monotherapy or Combined with Laser versus Laser Monotherapy in Asian Patients with Diabetic Macular Edema. Ophthalmology. 2015 Jul; 122(7):1402–15. doi: 10.1016/j.ophtha.2015.02.006 PMID: 25983216 | |
| RIDE | Nguyen QD, Brown DM, Marcus DM, et al. Ranibizumab for diabetic macular edema: results from 2 phase III randomized trials: RISE and RIDE. Ophthalmology. 2012;119:789–801. | |
| RISE | Nguyen QD, Brown DM, Marcus DM, et al. Ranibizumab for diabetic macular edema: results from 2 phase III randomized trials: RISE and RIDE. Ophthalmology. 2012;119:789–801. | |
| Soheilian2012 | Soheilian M, Garfami KH, Ramezani A, Yaseri M, Peyman GA. Two-year results of a randomized trial of intravitreal bevacizumab alone or combined with triamcinolone versus laser in diabetic macular edema. Retina (Philadelphia, Pa). 2012 Feb; 32(2):314–21. | |
| Shoeibi2013 | Shoeibi N, Ahmadieh H, Entezari M, Yaseri M. Intravitreal Bevacizumab with or without Triamcinolone for Refractory Diabetic Macular Edema: Long-term Results of a Clinical Trial. Journal of ophthalmic & vision research. 2013 Apr; 8(2):99–106. | |
| Synek2011 | Synek S, Veselý P. Intravitreal Bevacizumab with or without Triamcinolone for Refractory Diabetic Macular Oedema. Collegium antropologicum. 2011 Jul; 35(3):841–5. PMID: 22053565 | |
| Turkoglu2015 | Turkoglu EB, Celık E, Aksoy N, Bursalı O, Ucak T, Alagoz G. Changes in vision related quality of life in patients with diabetic macular edema: ranibizumab or laser treatment?. Journal of Diabetes and its Complications 2015;29(4):540-3. | |
| VISTA-DME | Korobelnik JF, Do DV, Schmidt-Erfurth U, Boyer DS, Holz FG, Heier JS, et al. Intravitreal aflibercept for diabetic macular edema. Ophthalmology. 2014;121:2247–54. | |
| VIVID-DME | Korobelnik JF, Do DV, Schmidt-Erfurth U, Boyer DS, Holz FG, Heier JS, et al. Intravitreal aflibercept for diabetic macular edema. Ophthalmology. 2014;121:2247–54. | |
| VIVID-EAST | Wells JA, Glassman AR, Ayala AR, et al. Aflibercept, bevacizumab, or ranibizumab for diabetic macular edema: two-year results from a comparative effectiveness randomized clinical trial. Ophthalmology. 2016; 123:1351–9. | |
| Wiley2016 | Wiley HE, Thompson DJ, Bailey C, Chew EY, Cukras CA, JaLe GJ, et al. A crossover design for comparative eLicacy: a 36-week randomized trial of bevacizumab and ranibizumab for diabetic macular edema. Ophthalmology 2016;123(4):841-9. | |
