# Supplementary material for: Diversity when interpreting evidence in network meta-analyses (NMAs) on similar topics: an example case of NMAs on diabetic macular oedema
Source: Syst Rev. 2023 Oct 7;12:189. doi: 10.1186/s13643-023-02349-4 (PMC10559427; doi:10.1186/s13643-023-02349-4)
Supplement: Supplementary file 3 — Additional file 3. Quality assessment by AMSTAR-2 Tool (Support information in detail). [file 13643_2023_2349_MOESM3_ESM.docx]

**Quality assessment by AMSTAR-2 Tool (Support information in detail)**

|  | **Item (* presents satisfied)** | **Korobelnik 2015** | **Re´gnier 2014** | **Zhang 2016** | **Muston 2018** | **Virgili 2018** |
| --- | --- | --- | --- | --- | --- | --- |
| **1. Did the research questions and inclusion criteria for the review include the components of PICO?** Yes/No | | Yes | Yes | No | Yes | Yes |
| For Yes | Population | * Quote:"Table 1 An overview of the PICOS and other criteria used for study inclusion and exclusion" (p3) | * Quote:"A systematic literature search was performed to identify relevant RCTs evaluating the efficacy of laser photocoagulation therapy, ranibizumab IVT injection, aflibercept IVT injection, ranibizumab plus laser or sham injection plus rescue laser therapy in the treatment of VI due to DME." (p2) | * Quote:"Participants with any type of diabetes" (p3) | * Comments: same to Korobelnik 2015. Quote:"Trials were screened using the populations, interventions, comparators, outcomes, and study design (pICOS) criteria as defined in the previous publication." (p2) | * Quote:"People with DMO for whom anti-VEGF treatment is indicated. We expected to include most of the studies also included in Virgili 2014." (p10) |
|  | Intervention | * Quote:"Table 1 An overview of the PICOS and other criteria used for study inclusion and exclusion" (p3) | * Quote:"...studies had to be RCTs that reported the outcome patients achieving a gain in BCVA of at least 10 letters (2 lines) on the ETDRS scale for at least two comparators of interest (sham injections plus rescue laser, ranibizumab 0.5 mg pro re nata [as needed], ranibizumab 0.5 mg pro re nata plus laser, aflibercept 2.0 mg bi-monthly [every 2 months] and prompt laser photocoagulation therapy)." (p2) | No information. | * Comments: same to Korobelnik 2015. Quote:"Trials were screened using the populations, interventions, comparators, outcomes, and study design (pICOS) criteria as defined in the previous publication." (p2) | * Quote:"Types of interventions" (p10) |
|  | Comparator group | * Quote:"Table 1 An overview of the PICOS and other criteria used for study inclusion and exclusion" (p3) | * Quote:"...studies had to be RCTs that reported the outcome patients achieving a gain in BCVA of at least 10 letters (2 lines) on the ETDRS scale for at least two comparators of interest (sham injections plus rescue laser, ranibizumab 0.5 mg pro re nata [as needed], ranibizumab 0.5 mg pro re nata plus laser, aflibercept 2.0 mg bi-monthly [every 2 months] and prompt laser photocoagulation therapy)." (p2) | No information. | * Comments: same to Korobelnik 2015. Quote:"Trials were screened using the populations, interventions, comparators, outcomes, and study design (pICOS) criteria as defined in the previous publication." (p2) | * Quote:"Types of interventions" (p10) |
|  | Outcome | * Quote:"Table 1 An overview of the PICOS and other criteria used for study inclusion and exclusion" (p3) | * Quote:"The outcome of interest was efficacy, assessed as the percentage of patients achieving a gain in BCVA of at least 10 letters (2 lines) on the Early Treatment Diabetic Retinopathy Study (ETDRS) scale. The outcome of interest was chosen following acceptance by NICE of a Markov model with 10-letter health states in the assessment of ranibizumab for DME. Changes in BCVA of 10 letters have been shown to be clinically significant in a number of studies." (p2) | * Quote:"BCVA, CMT, and ocular and systematic adverse events (AEs) were the primary endpoints of this meta-analysis." (p3) | * Comments: same to Korobelnik 2015. Quote:"Trials were screened using the populations, interventions, comparators, outcomes, and study design (pICOS) criteria as defined in the previous publication." (p2) | * Quote:"Types of outcome measures" (p10) |
| Optional (recommended) | Timeframe for follow-up | * Quote:"Table 1 An overview of the PICOS and other criteria used for study inclusion and exclusion" (p3) | * Quote:"The outcome of interest had to be measured at 6 or 12 months from study baseline, with 12 month data used for the analysis where available." (p3) | No information. | * Comments: same to Korobelnik 2015. Quote:"Trials were screened using the populations, interventions, comparators, outcomes, and study design (pICOS) criteria as defined in the previous publication." (p2) | * Quote:"Measurements at varying lengths of follow-up were pooled at annual intervals, plus or minus six months, the primary analysis being that at 12 months. The time point closer to 12 months, or the latest time point in the window frame in the case of symmetry, was chosen where multiple time points were available." (p10) |
| **2. Did the report of the review contain an explicit statement that the review methods were established prior to the conduct of the review and did the report justify any significant deviations from the protocol?** Yes/Partial Yes/No | | Partial Yes | No | No | No | Partial Yes |
| For Partial Yes: The authors state that they had a written protocol or guide that included ALL the following | review question(s) | * Quote:"The analysis approach was predefined in the study protocol." (4) Quote: "This review adhered to international recommendations and guidelines in order to reduce bias in publication selection, including pre-specification of inclusion/exclusion criteria and pre-specification of indirect comparisons of interest." (p12) Quote: "a number of strengths, including the adherence to international guidelines for performing indirect analyses, inclusion of prespecified inclusion and exclusion criteria, and comprehensive assessment of clinical and statistical heterogeneity" (p13) | No information. | No information. | No information. | * Quote:"Objective" (p10) |
|  | a search strategy | * Quote:"The analysis approach was predefined in the study protocol." (4) Quote: "This review adhered to international recommendations and guidelines in order to reduce bias in publication selection, including pre-specification of inclusion/exclusion criteria and pre-specification of indirect comparisons of interest." (p12) Quote: "a number of strengths, including the adherence to international guidelines for performing indirect analyses, inclusion of prespecified inclusion and exclusion criteria, and comprehensive assessment of clinical and statistical heterogeneity" (p13) | No information. | No information. | No information. | * Quote:"Search methods for identification of studies" (p10) |
|  | inclusion/exclusion criteria | * Quote:"The analysis approach was predefined in the study protocol." (4) Quote: "This review adhered to international recommendations and guidelines in order to reduce bias in publication selection, including pre-specification of inclusion/exclusion criteria and pre-specification of indirect comparisons of interest." (p12) Quote: "a number of strengths, including the adherence to international guidelines for performing indirect analyses, inclusion of prespecified inclusion and exclusion criteria, and comprehensive assessment of clinical and statistical heterogeneity" (p13) | No information. | No information. | No information. | * Quote:"Criteria for considering studies for this review" (p10) |
|  | a risk of bias assessment | * Quote:"The analysis approach was predefined in the study protocol." (4) Quote: "This review adhered to international recommendations and guidelines in order to reduce bias in publication selection, including pre-specification of inclusion/exclusion criteria and pre-specification of indirect comparisonsof interest." (p12) Quote: "a number of strengths, including the adherence to international guidelines for performing indirect analyses, inclusion of prespecified inclusion and exclusion criteria, and comprehensive assessment of clinical and statistical heterogeneity" (p13) | No information. | No information. | No information. | * Quote:"Assessment of risk of bias in included studies" (p11) |
| For Yes: As for partial yes, plus the protocol should be registered and should also have specified | a meta-analysis/synthesis plan, if appropriate | * Quote:"The analysis approach was predefined in the study protocol." (4) Quote: "This review adhered to international recommendations and guidelines in order to reduce bias in publication selection, including pre-specification of inclusion/exclusion criteria and pre-specification of indirect comparisons of interest." (p12) Quote: "a number of strengths, including the adherence to international guidelines for performing indirect analyses, inclusion of prespecified inclusion and exclusion criteria, and comprehensive assessment of clinical and statistical heterogeneity" (p13) | No information. | No information. | No information. | * Quote:"Data synthesis" (p12) |
|  | a plan for investigating causes of heterogeneity | * Quote:"The analysis approach was predefined in the study protocol." (4) Quote: "This review adhered to international recommendations and guidelines in order to reduce bias in publication selection, including pre-specification of inclusion/exclusion criteria and pre-specification of indirect comparisons of interest." (p12) Quote: "a number of strengths, including the adherence to international guidelines for performing indirect analyses, inclusion of prespecified inclusion and exclusion criteria, and comprehensive assessment of clinical and statistical heterogeneity" (p13) | No information. | No information. | No information. | No information. |
|  | justification for any deviations from the protocol | No information. | No information. | No information. | No information. | Quote:"Differences between protocol and review" (p142) |
| **3. Did the review authors explain their selection of the study designs for inclusion in the review?** Yes/No | | Yes | No | No | No | No |
| For Yes: the review should satisfy ONE of the following | Explanation for including only RCTs | Quote: Table 1 - "Pilot studies (if phase not mentioned), phase I and II randomized controlled studies (to be included as second-level evidence, if primary evidence is unavailable)" (p3) Quote: Table 1 - "Controlled observational studies (to be included as second-level evidence, if primary evidence is unavailable)" (p3) | No information. | No information. | No information. | No information. |
|  | OR Explanation for including only NRSI | Quote: Table 1 - "Pilot studies (if phase not mentioned), phase I and II randomized controlled studies (to be included as second-level evidence, if primary evidence is unavailable)" (p3) Quote: Table 1 - "Controlled observational studies (to be included as second-level evidence, if primary evidence is unavailable)" (p3) | No information. | No information. | No information. | No information. |
|  | OR Explanation for including both RCTs and NRSI | Quote: Table 1 - "Pilot studies (if phase not mentioned), phase I and II randomized controlled studies (to be included as second-level evidence, if primary evidence is unavailable)" (p3) Quote: Table 1 - "Controlled observational studies (to be included as second-level evidence, if primary evidence is unavailable)" (p3) | No information. | No information. | No information. | No information. |
| **4. Did the review authors use a comprehensive literature search strategy?** Yes/Partial Yes/No | | Yes | No | Partial Yes | Partial Yes | Partial Yes |
| For Partial Yes (all the following) | searched at least 2 databases (relevant to research question) | *  Quote:"The following databases were searched from inception: Medline (1946–2013/10); Medline In-Process Citations and Daily Update (up to 2013/10/13); Embase (1974– 2013/10); Cochrane Central Register of Controlled Trials (up to 2013/10/15)." (p2) | * Quote:"In Phase 1, recently published, relevant SRs with a low risk of bias were identified through searches of the electronic databases Embase, MEDLINE, MEDLINE In-Process and the Cochrane Library." (p2) | * Quote:"We searched and identified the relevant trials from PubMed, Embase and CENTRAL through August 2015 with no language limit" (p2) | * Comments: same to Korobelnik 2015. | * Quote:"Search methods for identification of studies" (p10) |
|  | provided key word and/or search strategy | * Quote:"Appendix 1, Appendix 2" (p2) | * Quote:"Table S1/2" (p2) | * Quote:"The medical subject heading and keywords used for the search included diabetic macular edema, laser, steroids, vascular endothelial growth factor, bev acizumab, ranibizumab, pegaptanib and aflibercept." (p2) | * Comments: same to Korobelnik 2015. | * Quote:"Appendix 1. CENTRAL search strategy" (p106) |
|  | justified publication restrictions (e.g. language) | * Quote:"Table 1 An overview of the PICOS and other criteria used for study inclusion and exclusion" (p4) | Comment: No justification as to why the search was restrcted to English, French and German. Quote:"Studies published in English, French and German were included." (p3) | * Quote:"We searched and identified the relevant trials from PubMed, Embase and CENTRAL through August 2015 with no language limit" (p2) | * Comments: same to Korobelnik 2015. | * Quote:"There were no language or publication year restrictions." (p10) |
| For Yes, should also have (all the following) | searched the reference lists / bibliographies of included studies | * Quote:"The bibliographies of identified research and review articles were also checked for studies." (p2) | * Quote:"In Phase 2, an additional search was conducted to identify any relevant RCTs published since the most recent identified SR." (p2) | * Quote:"We also screened the reference lists of pub lished meta-analyses of DME treatment." (p3) | * Comments: same to Korobelnik 2015. | * Quote:"We handsearched the reference lists of the included trials for other possible trials." (p10) |
|  | searched trial/study registries | * Quote:"A number of other searches were also undertaken, including other databases (rapid appraisal), websites, and congress abstracts, which are listed in Additional file 1: Appendix 2." (p2) | * Quote:"The third phase involved hand searching of abstracts from ophthalmology congresses (Association for Research in Vision and Ophthalmology [ARVO], American Academy of Ophthalmology [AAO] and European Society of Retina Specialists [EURETINA]), the ClinicalTrials.gov registry, and data on file at Novartis." (p2) | No information. | * Quote:"An additional search of ClinicalTrials.gov (from January 2015 to December 2016) was also performed to identify any new trials." (p2) | * Quote:"Search methods for identification of studies" (p10) |
|  | included/consulted content experts in the field | * Quote:"Additional data (including abstracts for any unpublished studies at the time of literature review) were provided by Bayer HealthCare (Berlin, Germany)." (p2) | No information. | No information. | No information. | No information. |
|  | where relevant, searched for grey literature | * Quote:"Additional data (including abstracts for any unpublished studies at the time of literature review) were provided by Bayer HealthCare (Berlin, Germany)." (p2) | * Quote:"...and data on file at Novartis." (p2) | No information. | * Comments: same to Korobelnik 2015. | No information. |
|  | conducted search within 24 months of completion of the review | * Quote: "The following databases were searched from inception: Medline (1946–2013/10); Medline In-Process Citations and Daily Update (up to 2013/10/13); Embase (1974–2013/10); Cochrane Central Register of Controlled Trials (up to 2013/10/15)." (p2) Comment: The study was published in 2015. So based on these two information provided, the search was conducted within 24 months of completion of the review. | * Comment: The study was published in 2014. The search was done till Feb 2014, so So based on the information provided, the search was conducted within 24 months of completion of the review | No information. | No information. | No information. |
| **5. Did the review authors perform study selection in duplicate?** Yes/No | | Yes | Yes | No | No | Yes |
| For Yes, either ONE of the following | at least two reviewers independently agreed on selection of eligible studies and achieved consensus on which studies to include | * Quote:"Titles and abstracts identified through the search strategies described were independently screened by two reviewers...Any discrepancies between reviewers were resolved through discussion or the intervention of a third reviewer." (p4) | * Quote:"Two authors (SR and FA) independently assessed the eligibility of all retrieved sources based on published abstracts. Non-relevant papers were excluded with the reasons for exclusion documented using a prospectively designed coding system. Discrepancies were resolved through discussion.Inclusion or exclusion of potentially relevant full-text RCT publications was then verified by three authors (FA, SR, WM) through a full text review" (p3) | No information. | No information. | * Quote:"Selection of studies" (p11) |
|  | OR two reviewers selected a sample of eligible studies and achieved good agreement (at least 80 percent), with the remainder selected by one reviewer | NA | NA | No information. | No information. | NA |
| **6. Did the review authors perform data extraction in duplicate?** Yes/No | | Yes | No | Yes | Yes | Yes |
| For Yes, either ONE of the following | at least two reviewers achieved consensus on which data to extract from included studies | * Quote:"A similar approach was undertaken for data extraction and quality assessment." (p4) | Comments: no information on how achieved consensus about data extraction. Quote:"Data were extracted by two authors (SR and WM)." (p3) Quote:"Two authors (SR and FA) independently assessed the quality of the selected studies, with discrepancies were resolved through discussion."(p3) | * Quote:"Investigators extracted data independently and made the final selection based on resolved discrepancies by discussion." (p3) | * Comments: same to Korobelnik 2015. Quote:"Data extraction was based on methods described previously." (p2) | * Quote:"Data extraction and management" (p11) |
|  | OR two reviewers extracted data from a sample of eligible studies and achieved good agreement (at least 80 percent), with the remainder extracted by one reviewer. | NA | No information. | NA | NA | NA |
| **7. Did the review authors provide a list of excluded studies and justify the exclusions?** Yes/Partial Yes/No | | No | Yes | No | No | Yes |
| For Partial Yes | provided a list of all potentially relevant studies that were read in full-text form but excluded from the review | Comments: this describes only studies that were further excluded from the list of 75 included studies based on whether they provide relevant data for indirect analysis. This study report should have provided a list of studies along with reasons for exclusion for the 165 (125+40) studies excluded for reasons of PICOS. It did not. | * Quote:"Of these, seven were excluded because they included either no or only one regimen of interest (RELIGHT, RETAIN, READ-3, RaScaL, OPTIMAL, RIDE and RISE), one (RED-ES) was excluded as it did not have any available results, and one (REVEAL) was excluded from the base-case analysis (but included in sensitivity analyses) because the focus was on a single ethnic group (Asian)." (p4) | Comments: authors did not provide a list of excluded studies. Quote:"Literature Search" (p3) | Comments: authors did not provide a list of excluded studies. Quote:"Literature Search" (p3) | * Quote:"Characteristics of excluded studies" (p83) |
| For Yes, must also have | Justified the exclusion from the review of each potentially relevant study | Comments: this describes only studies that were further excluded from the list of 75 included studies based on whether they provide relevant data for indirect analysis. This study report should have provided a list of studies along with reasons for exclusion for the 165 (125+40) studies excluded for reasons of PICOS. It did not. | * Quote:"Table S3. Studies from randomized controlled trial database search excluded based on full text review." (p4) | No information. | No information. | * Quote:"Characteristics of excluded studies" (p83) |
| **8. Did the review authors describe the included studies in adequate detail?** Yes/Partial Yes/No | | Partial Yes | Partial Yes | Partial Yes | Partial Yes | Partial Yes |
| For Partial Yes (ALL the following) | described populations | * Comments: authors described number of patients in each trail. Quote:"Table 2 An overview of the studies (n = 11) included in the final analyses" (p8) | * Comments: authors described number of patients in each trail. Quote:"Table 1. Summary of the proportion of patients gaining more than 10 letters (BCVA) from baseline to month 12 by study and treatment group." (p6) | * Comments: authors described number of eyes. Quote:"Table 1. " (p4) | * Comments: authors described number of patients in each trail. Quote:" an overview of the outcomes reported in these trials is summarised in Additional file 3: Appendix 3. " (p4) | * Comments: authors described number of patients, sex and age. Quote:"Characteristics of included studies" (p36) |
|  | described interventions | * Comments: authors described dosage and regiment of anti-vegf. Quote:"Appendix 4 Treatment regimens of included studies." (p5) | * Comments: authors described dosage and regiment of anti-vegf. Quote:"Table 1. Summary of the proportion of patients gaining more than 10 letters (BCVA) from baseline to month 12 by study and treatment group." (p6) | * Comments: authors described dose of intravitreal anti-vegf. Quote:"Table 1. " (p4) | * Comments: authors described dosage and regiment of anti-vegf. Quote:"The treatment regimens in these trials were classified and included in the NMA as summarised in Additional file 2: Appendix 2" (p4) | * Comments: authors described intravitreal anti-vegf dosage and regiments. Quote:"Characteristics of included studies" (p36) |
|  | described comparators | * Comments: authors described dosage and regiment of anti-vegf, steroid and slaser. Quote:"Appendix 4 Treatment regimens of included studies." (p5) | * Comments: authors described dosage and regiment of anti-vegf. Quote:"Table 1. Summary of the proportion of patients gaining more than 10 letters (BCVA) from baseline to month 12 by study and treatment group." (p6) | * Comments: authors described dose of intravitreal anti-vegf. Quote:"Table 1. " (p4) | * Comments: authors described dosage and regiment of anti-vegf, steroid and slaser. Comments: authors described dosage and regiment of anti-vegf. Quote:"The treatment regimens in these trials were classified and included in the NMA as summarised in Additional file 2: Appendix 2" (p4) | * Comments: authors did not described the regiment in each trail. Quote:"Characteristics of included studies" (p36) |
|  | described outcomes | * Comments: authors stated primary outcome in each trail. Quote:"Table 2 An overview of the studies (n = 11) included in the final analyses" (p8) | * Comments: authors described BCVA and CRT in each trail. Quote:"Table 1. Summary of the proportion of patients gaining more than 10 letters (BCVA) from baseline to month 12 by study and treatment group." (p6) Quote:"Baseline BCVA and CRT of patient populations varied among the included RCTs (Table S6 and Table S7)." (p6) | Comments: authors descriped BCVA and CMT in each trail. Quote:"Table 1. " (p4) | * Quote:"...an overview of the outcomes reported in these trials is summarised in Additional file 3: Appendix 3. " (p4) | * Comments: authors descriped all of outcomes in each trail. Quote:"Characteristics of included studies" (p36) |
|  | described research designs | * Quote:"Table 2 An overview of the studies (n = 11) included in the final analyses" (p8) | * Quote:"Overall, eight RCTs were included in the base-case analysis (VIVID, VISTA, DA VINCI, RESTORE, READ-2, RESOLVE, RESPOND, DRCR.net Protocol I." (p4) | * Quote:"The 21 eligible RCTs contained a total of 4307 eyes, including 428 eyes with 6-month follow-up and 3879 eyes with 12-month follow-up." (p7) | * Quote:"A total of 13 trials were identified as eligible for inclusion." (p4) | * Comments: authors descriped method in each trail. Quote:"Characteristics of included studies" (p36) |
| For Yes, should also have ALL the following | described population in detail | Comments: authors described number of patients in each trail. Quote:"Table 2 An overview of the studies (n = 11) included in the final analyses" (p8) | Comments: authors described number of patients in each trail. Quote:"Table 1. Summary of the proportion of patients gaining more than 10 letters (BCVA) from baseline to month 12 by study and treatment group." (p6) | Comments: authors described number of eyes. Quote:"Table 1. " (p4) | Comments: authors described number of patients in each trail. Quote:" an overview of the outcomes reported in these trials is summarised in Additional file 3: Appendix 3. " (p4) | * Comments: authors described number of patients, sex and age. Quote:"Characteristics of included studies" (p36) |
|  | described intervention in detail (including doses where relevant) | * Comments: authors described dosage and regiment of anti-vegf. Quote:"Appendix 4 Treatment regimens of included studies." (p5) | * Comments: authors described dosage and regiment of anti-vegf. Quote:"Table 1. Summary of the proportion of patients gaining more than 10 letters (BCVA) from baseline to month 12 by study and treatment group." (p6) | Comments: authors described dose of intravitreal anti-vegf. Quote:"Table 1. " (p4) | * Comments: authors described dosage and regiment of anti-vegf. Quote:"The treatment regimens in these trials were classified and included in the NMA as summarised in Additional file 2: Appendix 2" (p4) | * Comments: authors described intravitreal anti-vegf dosage and regiments. Quote:"Characteristics of included studies" (p36) |
|  | described comparator in detail (including doses where relevant) | * Comments: authors described dosage and regiment of anti-vegf, steroid and slaser. Quote:"Appendix 4 Treatment regimens of included studies." (p5) | * Comments: authors described dosage and regiment of anti-vegf. Quote:"Table 1. Summary of the proportion of patients gaining more than 10 letters (BCVA) from baseline to month 12 by study and treatment group." (p6) | Comments: authors described dose of intravitreal anti-vegf. Quote:"Table 1. " (p4) | * Comments: authors described dosage and regiment of anti-vegf, steroid and slaser. Comments: authors described dosage and regiment of anti-vegf. Quote:"The treatment regimens in these trials were classified and included in the NMA as summarised in Additional file 2: Appendix 2" (p4) | Comments: authors did not described the regiment of laser in each trail. |
|  | described study’s setting | No information. | No information. | No information. | No information. | No information. |
|  | timeframe for follow-up | * Quote:"These studies are summarized in Additional file 1: Appendix 3." (p5) | * Quote:"Table 1. Summary of the proportion of patients gaining more than 10 letters (BCVA) from baseline to month 12 by study and treatment group." (p6) | * Quote:"The 21 eligible RCTs contained a total of 4307 eyes, including 428 eyes with 6-month follow-up and 3879 eyes with 12-month follow-up." (p7) | * Comments: only trials that reported the randomised controlled results at 12 months were included. Quote:"A total of 13 trials were identified as eligible for inclusion." (p4) | * Comments: authors descriped follow-up time in each trail. Quote:"Characteristics of included studies" (p36) |
| **9. Did the review authors use a satisfactory technique for assessing the risk of bias (RoB) in individual studies that were included in the review?** Yes/Partial Yes/No/Includes only NRSI/Includes only RCTs | | Yes | Partial Yes | Yes | No | Yes |
| **RCTs** For Partial Yes, must have assessed RoB from | unconcealed allocation | * Quote:"Appendix 5 Summary of the risk of bias" (p7) | * Quote:"Overall the studies were of good quality, although some issues were identified (Table S5)." (p4) | * Quote:"Risk of Bias" (p7) | No information. | * Quote:"Characteristics of included studies [ordered by study ID]" (p36) |
|  | and lack of blinding of patients and assessors when assessing outcomes (unnecessary for objective outcomes such as all-cause mortality) | * Quote:"Appendix 5 Summary of the risk of bias" (p7) | * Quote:"Overall the studies were of good quality, although some issues were identified (Table S5)." (p4) | * Quote:"Risk of Bias" (p7) | No information. | * Quote:"Characteristics of included studies [ordered by study ID]" (p36) |
| **RCTs** For Yes, must also have assessed RoB from | allocation sequence that was not truly random | * Quote:"Appendix 5 Summary of the risk of bias" (p7) | * Quote:"Overall the studies were of good quality, although some issues were identified (Table S5)." (p4) | * Quote:"Risk of Bias" (p7) | No information. | * Quote:"Characteristics of included studies [ordered by study ID]" (p36) |
|  | and selection of the reported result from among multiple measurements or analyses of a specified outcome | * Quote:"Appendix 5 Summary of the risk of bias" (p7) | Comments: authors only assessed if any evidence to suggest that the authors measured more outcomes than they reported, but did not assessed if any evidence to suggest that the authors measured less outcomes than they reported. Quote:"Overall the studies were of good quality, although some issues were identified (Table S5)." (p4) | * Quote:"Risk of Bias" (p7) | No information. | * Quote:"Characteristics of included studies [ordered by study ID]" (p36) |
| **NRSI** For Partial Yes, must have assessed RoB | from confounding | NA | NA | NA | NA | NA |
|  | and from selection bias | NA | NA | NA | NA | NA |
| **NRSI** For Yes, must also have assessed RoB | methods used to ascertain exposures and outcomes | NA | NA | NA | NA | NA |
|  | and selection of the reported result from among multiple measurements or analyses of a specified outcome | NA | NA | NA | NA | NA |
| **10. Did the review authors report on the sources of funding for the studies included in the review?** Yes/No | | No | No | No | No | Yes |
| For Yes | Must have reported on the sources of funding for individual studies included in the review. Note: Reporting that the reviewers looked for this information but it was not reported by study authors also qualifies | No information. | No information. | No information. | No information. | * Quote:"Characteristics of included studies" (p36) |
| **11. If meta-analysis was performed did the review authors use appropriate methods for statistical combination of results?** Yes/No/No meta-analysis conducted | | Yes | No | No | No | Yes |
| **RCTs** For Yes | The authors justified combining the data in a meta-analysis | * Quote:"Statistical analyses" (p4) | * Quote:"Sensitivity analyses" (p3) | * Comments: 11 different interventions in NMA. | * Quote:"Analyse" (p3) | * Quote:"These two ranibizumab doses were merged into one group in our NMA since studies suggest no diLerence between them when used monthly (Heier 2016)." (p10) |
|  | AND they used an appropriate weighted technique to combine study results and adjusted for heterogeneity if present | Comments: no information about adjusted for heterogeneity if present. | Comments: no information about adjusted for heterogeneity if present. | Comments: no information about adjusted for heterogeneity if present. Quote:"Statistical Analysis" (3) | Comments: no information about adjusted for heterogeneity if present. | * Quote:"Methods for direct treatment comparisons" (p11) |
|  | AND investigated the causes of any heterogeneity | No information. | No information. | Comments: authors did not investigate the causes of any heterogeneity. Quote:"However, there was significant heterogeneity in the comparison between IVB and LASER." (p8) | No information. | * Quote:"Statistical heterogeneity between studies" (p25) |
| **For NRSI** For Yes | The authors justified combining the data in a meta-analysis | NA | NA | NA | NA | NA |
|  | AND they used an appropriate weighted technique to combine study results, adjusting for heterogeneity if present | NA | NA | NA | NA | NA |
|  | AND they statistically combined effect estimates from NRSI that were adjusted for confounding, rather than combining raw data, or justified combining raw data when adjusted effect estimates were not available | NA | NA | NA | NA | NA |
|  | AND they reported separate summary estimates for RCTs and NRSI separately when both were included in the review | NA | NA | NA | NA | NA |
| **12. If meta-analysis was performed, did the review authors assess the potential impact of RoB in individual studies on the results of the meta-analysis or other evidence synthesis?** Yes/No/No meta-analysis | | No | Yes | No | No | Yes |
| For Yes | included only low risk of bias RCTs | No information. | NA | No information. | No information. | NA |
|  | OR, if the pooled estimate was based on RCTs and/or NRSI at variable RoB, the authors performed analyses to investigate possible impact of RoB on summary estimates of effect. | No information. | * Quote:"In a scenario excluding READ-2, the efficacy of ranibizumab monotherapy remained numerically, but not statistically significantly superior to aflibercept monotherapy (OR, 1.42; 95% CrI, 0.30–8.71)." (p7) | No information. | No information. | * Quote:"We conducted analyses of efficacy outcomes excluding 10 studies at unclear or high risk of bias." (p26) |
| **13. Did the review authors account for RoB in individual studies when interpreting/ discussing the results of the review?** Yes/No | | No | Yes | No | No | Yes |
| For Yes | included only low risk of bias RCTs | No information. | NA | No information. | No information. | NA |
|  | OR, if RCTs with moderate or high RoB, or NRSI were included the review provided a discussion of the likely impact of RoB on the results | No information. | * Quote:" The analysis included a relatively small number of RCTs, of which three are not yet published in full and although the RCTs included in the metaanalysis were, in general, of good quality, the use of masking was not clearly reported in READ-2. Exclusion of READ-2 during sensitivity analyses suggested that this did not have a substantial effect on the results." (p8) | No information. | No information. | * Quote:"We conducted analyses of eLicacy outcomes aEer excluding 10 studies at unclear or high risk of bias." (p26) |
| **14. Did the review authors provide a satisfactory explanation for, and discussion of, any heterogeneity observed in the results of the review?** Yes/No | | No | No | No | No | Yes |
| For Yes | There was no significant heterogeneity in the results | Comments: authors did not investigate the causes of any heterogeneity. Quote:"there was high heterogeneity for all non-ocular AEs (I2 = 86%)" (p10) | No information. | Comments: authors did not investigate the causes of any heterogeneity. Quote:"However, there was significant heterogeneity in the comparison between IVB and LASER (p = 0.04, S1 Table)." (p8) | No information. | NA |
|  | OR if heterogeneity was present the authors performed an investigation of sources of any heterogeneity in the results and discussed the impact of this on the results of the review | Comments: authors did not investigate the causes of any heterogeneity. Quote:"there was high heterogeneity for all non-ocular AEs (I2 = 86%)" (p10) | No information. | Comments: authors did not investigate the causes of any heterogeneity. Quote:"However, there was significant heterogeneity in the comparison between IVB and LASER." (p8) | No information. | * Quote:"Statistical heterogeneity between studies" (p25) |
| **15. If they performed quantitative synthesis did the review authors carry out an adequate investigation of publication bias (small study bias) and discuss its likely impact on the results of the review?** Yes/No/No meta-analysis conducted | | No | No | No | No | Yes |
| For Yes | performed graphical or statistical tests for publication bias and discussed the likelihood and magnitude of impact of publication bias | No information. | No information. | No information. | NA | * Quote:"Selective reporting" (p26) |
| **16. Did the review authors report any potential sources of conflict of interest, including any funding they received for conducting the review?** Yes/No | | Yes | Yes | Yes | Yes | Yes |
| For Yes | The authors reported no competing interests OR | NA | NA | Quote:” The authors have declared that no competing interests exist.” | NA | NA |
|  | The authors described their funding sources and how they managed potential conflicts of interest | Quote:"The authors have the following financial competing interests to declare: Jos Kleijnen, Shona H. Lang, Richard Birnie, Regina M. Leadley, Kate Misso and Gill Worthy are employees of Kleijnen Systematic Reviews, which was funded by Bayer Pharma AG to undertake the project on which this paper is based. Dominic Muston is an employee of Bayer. Jean-Francois Korobelnik is a consultant for Alcon, Allergan, Bayer, Novartis, Roche, Thea, and Zeiss. Diana V. Do is a consultant for Bayer, Genentech, Regeneron, and Allergan." (p13) | Quote:"Funding: This study was funded by Novartis Pharma AG. The funder provided support in the form of salaries for authors SR, WM, FA and VB, but did not have any additional role in the study design, data collection and analysis, decision to publish, or preparation of the manuscript. The specific roles of these authors are articulated in the ‘author contributions’ section. Novartis Pharma AG and Genentech, Inc. reviewed the manuscript before submission. Third-party medical writing assistance, but not editorial content sufficient to meet International Committee of Medical Journal Editors (ICMJE) authorship criteria, was funded by Novartis Pharma AG." Quote:"Competing Interests: All authors have read the Journal’s submission policy and declare the following competing interests: Stephane Regnier and Vladimir Bezlyak are employees of Novartis Pharma AG. William Malcolm and Felicity Allen are employees of Novartis Pharmaceuticals UK Ltd. William Malcolm, Felicity Allen and Vladimir Bezlyak own Novartis shares. Novartis has exclusive rights to ranibizumab outside the United States of America and Novartis Pharma AG. funded this study. Jonathan Wright is an employee of Numerus Ltd. Numerus Ltd was paid to conduct analyses for this manuscript. There are no further patents, products in development or marketed products to declare. This does not alter the authors’ adherence to all the PLOS ONE policies on sharing data and materials, as detailed online in the guide for authors." (p1) | Quote:" This study was partly supported by a grant from the National Natural Science Foundation of China (81570820)." | Quote:"Competing interests" (p10) The authors have the following financial competing interests to declare: Dominic Muston is a former employee of Bayer. Jean-Francois Korobelnik is a consultant for Alcon, Allergan, Bayer, Kanghong, Novartis, and Roche. Tim Reason, Ismini Chatzitheofilou, and Fay Ryan are employees of QuintilesIMS, which was funded by Bayer to undertake the project on which this article is based. Neil Hawkins is a consultant for Bayer. Peter Kaiser is a consultant for Alcon, Allergan, Bayer, Kanghong, Novartis, and Regeneron.  “The analysis was funded by Bayer Pharma AG, who were involved in the study design, data collection, data analysis, and manuscript writing.” | Quote:"Declarations of interest" (p142)  “Gianni Virgili: none known Mariacristina Parravano received payment for participating on the Advisory Board for Allergan, Bayer and Novartis. Jennifer Evans: none known Iris Gordon: none known Ersilia Lucenteforte: none known  “S O U R C E S O F S U P P O R T  Internal sources  • Azienda Ospedaliero-Universitaria Careggi & University of Florence, based on funding by the Tuscany Region, Italy.  External sources  • National Institute for Health Research (NIHR), UK.  * Richard Wormald, Co-ordinating Editor for Cochrane Eyes and Vision (CEV) acknowledges financial support for his CEV research sessions from the Department of Health through the award made by the NIHR to Moorfields Eye Hospital NHS Foundation Trust and UCL Institute of Ophthalmology for a Specialist Biomedical Research Centre for Ophthalmology.  * This review was supported by the NIHR, via Cochrane Infrastructure funding to the CEV UK editorial base.  * The Cochrane Review Incentive Scheme provided funding for Jennifer Evans to assist with the 2014 update of this review.  The views and opinions expressed therein are those of the authors and do not necessarily reflect those of the Systematic Reviews  Programme, NIHR, NHS or the Department of Health. |
